# Immunotherapy for recurrent or metastatic nasopharyngeal carcinoma

**DOI:** 10.1038/s41698-024-00601-1

**Published:** 2024-05-16

**Authors:** Xin Liu, Hui Shen, Lu Zhang, Wenhui Huang, Shuixing Zhang, Bin Zhang

**Affiliations:** 1 The First Affiliated Hospital of Jinan University, Guangzhou, Guangdong China; 2 Graduate College, Jinan University, Guangzhou, Guangdong China

**Keywords:** Cancer immunotherapy, Immunotherapy

## Abstract

Immunotherapy, particularly immune checkpoint inhibitors (ICIs), such as anti-programmed death 1/programmed death-ligand 1 (PD-1/PD-L1) therapy, has emerged as a pivotal treatment modality for solid tumors, including recurrent or metastatic nasopharyngeal carcinoma (R/M-NPC). Despite the advancements in the utilization of ICIs, there is still room for further improving patient outcomes. Another promising approach to immunotherapy for R/M-NPC involves adoptive cell therapy (ACT), which aims to stimulate systemic anti-tumor immunity. However, individual agent therapies targeting dendritic cells (DCs) appear to still be in the clinical trial phase. This current review underscores the potential of immunotherapy as a valuable adjunct to the treatment paradigm for R/M-NPC patients. Further research is warranted to enhance the efficacy of immunotherapy through the implementation of strategies such as combination therapies and overcoming immune suppression. Additionally, the development of a biomarker-based scoring system is essential for identifying suitable candidates for precision immunotherapy.

## Introduction

Nasopharyngeal carcinoma (NPC) is particularly prevalent in East and Southeast Asia^1^. Unlike other subtypes of head and neck squamous cell carcinomas (HNSCCs), NPC is distinguished by its non-surgical treatment modality, etiology, and prognosis. Notably, trials that have led to the approval of promising immunotherapies for HNSCCs, such as nivolumab and pembrolizumab, have specifically excluded NPC patients from their study cohorts^2,3^. Among HNSCCs, NPC stands out as one of the most prone to recurrence and distant metastasis^4^, contributing to the limited clinical benefits and unfavorable prognosis associated with this cancer type. Patients with recurrent or metastatic NPC (R/M-NPC) are typically recommended to receive platinum-based chemotherapy as the first-line treatment. However, gemcitabine plus cisplatin (GP) therapy, while commonly used in recent years, provides only modest short-term benefits, with a 12-month progression-free survival (PFS) rate of only 20% in R/M NPC patients^5^. Immunotherapy has emerged as a transformative approach to cancer treatment, revolutionizing strategies for various types of cancer. In this review, we will focus on clinically approved immunotherapy regimens for R/M-NPC therapy and discuss novel immunotherapy strategies, challenges, and future directions for Immunotherapy combination strategies in R/M-NPC.

## Tumor immune microenvironment and targets for immunotherapy

**Fig. 1:**
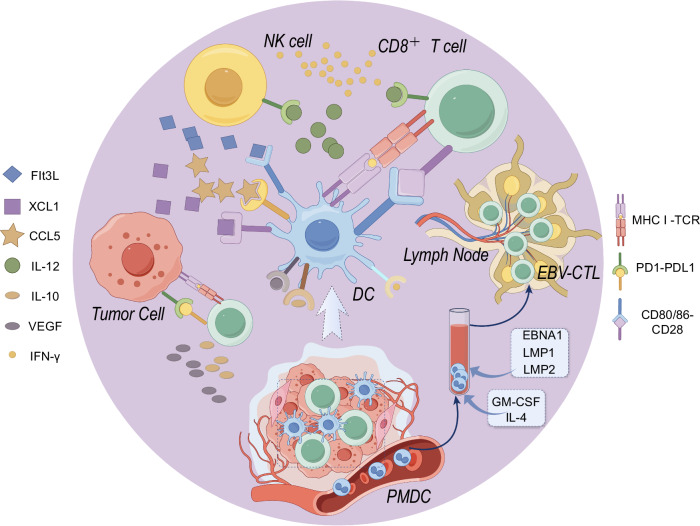
The molecular mechanisms of immunotherapy strategies and the interaction between anti-tumor immune cells and the tumor immune microenvironment. PMDC Peripheral blood mononuclear cell, DC Dendritic cell, NK cell nature killer cell, EBV-CTL EBV-specific cytotoxic T lymphocyte, EBNA1 EBV nuclear antigen 1, LMP1 and LMP2 Latent membrane proteins 1 and 2, GM-CSF Granulocyte-macrophage colony-stimulating factor, IL-4 Interleukin-4, Flt3L FMS-related tyrosine kinase 3 ligand, XCL1 X-C motif chemokine ligand 1, CCL5 C-C motif chemokine ligand 5, IL-12: Interleukin-12, IL-10 Interleukin-10, VEGF Vascular endothelial growth factor, IFN-γ interferon-γ, MHC Major histocompatibility complex, TCR T cell receptor. (By Figdraw.).

NPC is closely associated with the Epstein-Barr virus (EBV) infection, characterized by significant lymphocytic infiltrations in tumor tissues and heightened programmed death-ligand 1 (PD-L1) expression^6^. With advancements in understanding the tumor immune microenvironment (TIME), there is a growing emphasis on investigating the systemic anti-tumor immune responses, wherein dendritic cells (DCs) and CD8 T cells play pivotal roles^7^. CD8 T cells are known to be crucial in facilitating anti-tumor immunity through the recognition of tumor-related antigens presented on major histocompatibility complexes class (MHC) I via their T cell receptor (TCR). Moreover, specialized antigen-presenting cells, particularly DCs, are essential for the activation and maintenance of CD8 T cells’ cytotoxic immune responses. The interaction between co-stimulatory molecules CD80/CD86 and CD28 provides a secondary signal for T cell activation. Additionally, natural killer cells produce either FMS-related tyrosine kinase 3 ligand or C-C motif chemokine ligand 5 and X-C motif chemokine ligand 1, which induces the recruitment of DCs into the TIME. Bidirectional communication has also been demonstrated as necessary, with the production of interleukin-12 (IL-12) by DCs leading to the production of interferon-γ (IFN-γ) by CD8 T cells and natural killer cells. In summary, these processes highlight the beneficial aspects of cross-presenting tumor antigens to naive CD8 T cells in lymph nodes to induce successful anti-tumor immune responses. Conversely, within the TIME, NPC tumor cells hinder the function of DCs by secreting negative regulatory factors, such as IL-10 and vascular endothelial growth factor. Simultaneously, the high expression of PD-L1 on the surface of NPC tumor cells, and its binding to the inhibitory protein PD-1 produced by CD8 T cells, enable evasion of the adaptive immune system through down-regulation of T-cell response^7–10^ (Fig. 1).

Immunotherapy strategies can be broadly categorized into two main groups. The first group aims to enhance the existing adaptive immune response by inhibiting inhibitory pathways on T cells within the tumor. A common example of this approach is the use of immune checkpoint inhibitors (ICIs) targeting PD-1. The second group encompasses active immunotherapy methods such as DC vaccines, adoptive cell transfer of tumor-specific T cells, and engineered T cells with tumor antigen-specific T cell receptors or chimeric antigen receptors^9–11^.

## Immune checkpoint inhibitors for R/M-NPC

Several studies have suggested a close association between elevated PD-L1 on NPC tumor cells and poor prognosis among patients undergoing traditional radiotherapy and chemotherapy^12,13^. This suggests that anti-PD-1/PD-L1 therapy could be a promising treatment approach and a means to improve patients’ prognosis.

In recent years, the emergence of PD-1 antibodies has presented a promising avenue for immunotherapeutic intervention in the management of R/M-NPC. In an international, double-blind, randomized, placebo-controlled phase 3 trial (JUPITER-02)^14^, Mai et al. compared the efficacy and toxicity of GP chemotherapy combined with either toripalimab or placebo as a first-line treatment for patients with R/M NPC. A total of 289 eligible patients from mainland China, Taiwan, and Singapore were evenly distributed between the toripalimab combination arm (arm A, *n* = 146) and the placebo combination arm (arm B, *n* = 143). The median PFS was 11.7 months in the toripalimab arm and 8.0 months in the placebo arm. The results showed that treatment with toripalimab in combination with chemotherapy reduced the risk of progression or death by 59% compared to placebo combined with chemotherapy (hazard ratio [HR] = 0.41, *P* < 0.0001), while maintaining a manageable safety profile. Furthermore, their findings suggested that the clinical benefits of the toripalimab-GP combination could be observed irrespective of PD-L1 expression status. In 2021, Yang et al. conducted a multicenter, randomized, double-blind, phase 3 trial (CAPTAIN-1st)^15^ to compare the clinical efficacy of camrelizumab plus GP versus placebo plus GP as a first-line treatment for R/M-NPC. A total of 263 eligible patients were randomly assigned to receive either camrelizumab (*n* = 134) or placebo (*n* = 129) plus GP. The study revealed a significantly longer PFS in the camrelizumab group compared to the placebo group (median, 9.7 vs 6.9 months; one-sided *P* = 0.0002). Furthermore, the safety profiles of camrelizumab plus chemotherapy were found to be manageable.

However, there is currently no established standard for salvage therapy plans for patients who have experienced treatment failure with initial platinum-containing regimens. In recent years, several anti-PD-1 monoclonal antibodies have emerged as potential options for salvage treatment following initial standard interventions for locally advanced NPC, with individual response rates ranging from 20% to 30%^16–19^. Among these trials, the most impactful is a phase II clinical trial (POLARIS-02) conducted by the Sun Yat-sen University Cancer Center^18^. This trial demonstrated an overall response rate (ORR) of 20.5%, with a median disease control rate (DOR) of 12.8 months, median PFS of 1.9 months, and a median overall survival (OS) of 17.4 months among patients with R/M-NPC who received toripalimab monotherapy as second-line and beyond therapy. In this systematic review, we conducted a meta-analysis to evaluate the association between second-line and subsequent treatments using anti-PD-1 antibodies and the primary endpoints in R/M-NPC, which include ORR, DCR, PFS, and OS. Table 1 presents a summary of the characteristics of the included 12 studies (including one randomized controlled trial and 11 retrospective cohort studies). The pooled analysis utilizing the random-effects model yielded an ORR of 23% (95% CI: 18-28%, I^2^ = 52%) and a DCR of 60% (95% CI: 49-71%, I^2^ = 95%) when anti-PD1 drugs were administered as second-line and subsequent treatments in R/M-NPC patients (Supplementary Figure 2). Detailed methods and results of the meta-analysis are provided in the Supplementary Material.

**Table 1:** Characteristics of the included studies on immune checkpoint inhibitors for R/M-NPC

Study	Study design	Population	Experimental arm	Dosing regimen	Sample size (n)	Mean age (years)	Male n/(%)	Outcome measures	Median PFS (months)	Median OS (months)	Any AEs n (%)	High grade AEs n (%)
Hsu 2017	Non-Randomize, multicohort, phase Ib trial	R/M-NPC	pembrolizumab	10 mg/kg was administered intravenously once every 2 weeks for 24 months	27	52	21 (77.8%)	CR, PR, SD, PD; ORR, DCR	6.5 (95% CI: 3.6–13.4) 6-month PFS rate, 50.0% 1-year PFS rate, 33.4%	16.5 (95% CI: 10.1 to not reached) 6-month OS rate 85.2% 1-year OS rate 63.0%	20 (74.1%)	8 (29.6%)
Ma 2018	Retrospective cohort	R/M-NPC	Nivolumab	3 mg/kg intravenously every 2 weeks on a 4-week cycle	45	57	35 (77.8%)	CR, PR, SD, PD; ORR, DCR	2.8 (95% CI: 1.8–7.4) 1-year PFS rate, 19.3%	17.1 (95% CI: 10.9 to not reached) 1-year OS rate, 59%	NS	NS
Fang 2018	Single-arm, phase 1 trials	R/M-NPC	Camrelizumab	The prespecified doses of 1 mg/kg, 3 mg/kg, and 10 mg/kg, intravenously over 30 min once every 2 weeks	93	45	75 (81%)	CR, PR, SD, PD; ORR, DCR	5.6 (95%CI: 3.3–7.9) 6-month PFS rate, 48.2% 1-year PFS rate, 27.1%	NS	90 (97%)	8 (9%)
Ma 2019	Single arm trial 1/2	R/M-NPC	Nivolumab	3 mg/kg, once every 2 weeks	32	NS	N.S	CR, PR,SD, PD; ORR, DCR	NS	NS	22 (69%)	1 (3%)
Sato H 2020	Retrospective cohort	R/M-NPC	Nivolumab	3 mg/kg every 2 weeks,and subsequently 240 mg every 2 weeks	12	58	10 (83.3%)	CR, PR, SD, PD; ORR, DCR	NS	NS	8 (66.7%)	1 (8.3%)
Shen 2020	Retrospective cohort	R/M-NPC	Tislelizumab	Tislelizumab 200 mg once every 3 weeks	21	NS	N.S	CR, PR, SD, PD; ORR, DCR	NS	NS	NS	NS
Kim 2020	Retrospective cohort	R/M-NPC	Nivolumab	200 mg Pembrolizumab once every 3 weeks	8	47.4	N.S	CR, PR, SD, PD; ORR, DCR	NS	N.S	NS	NS
Wang 2021	Single-arm, phase II Study	R/M-NPC	Toripalimab	3 mg/kg once every 2 weeks via intravenous infusion	190	46.4	158 (83.2%)	CR, PR, SD, PD; ORR, DCR	1.9 (95% CI: 1.8–3.5)	17.4 (95%CI: 11.7- 22.9)	141 (74.2%)	27 (14.2%)
Yang 2021	Single-arm, phase II Study	R/M-NPC	Camrelizumab	200 mg intravenously every 2 weeks on 4-week treatment cycles	156	48	124 (79.5%)	CR, PR, SD, PD ORR, DCR	3.7 (95% CI: 2.0–4.1)	17.4 (95%CI: 15.2-21.9)	155 (99.4%)	52 (33.3%)
Even 2021	Randomized Controlled, phase II Study	R/M- NPC	Spartalizumab	400 mg every 4 weeks in 28-day cycles	82	51	68 (82.9%)	CR, PR, SD, PD; ORR, DCR	1.9 (95% CI: 1.8–3.6)	25.2 (95% CI: 13.1 to NE)	59 (72.0%)	14 (17.1%)
Jin 2021	Non-randomised hypothesis generatings	R/M-NPC	Anti-PD1 checkpoint inhibitor monotherapy	200 mg Camrelizumab on Day 1 every 2/3 weeks, 240 mg Toripalimab on day 1 every 3 weeks, 200 mg Penpulimab on day 1 every 2 weeks or 200 mg Tisleizumab on day 1 every 3 weeks	41	<60: 73.2% ≥ 60: 26.8%	28 (68.3%)	CR, PR, SD, PD; ORR, DCR	NS	NS	41 (100%)	6 (14.6%)
Jung 2022	Single-arm, phase II Study	R/M-NPC	Nivolumab plus gemcitabine	nivolumab (3 mg/kg) and gemcitabine (1250 mg/m2) every 2 weeks	36	50.5	30 (83.3%)	CR, PR, SD, PD; ORR, DCR	6-month PFS rate, 70.3% 1-year PFS rate, 60.5%	6-month OS rate, 97.0% 1-year OS rate, 87.7%	NS	2 (5.6%)

*NS* Not Stated, *R/M NPC* recurrent and metastatic nasopharyngeal carcinoma, *ORR* Overall Response Rate, *CR* Complete Response, *PR* Partial Response, *SD* Stable Disease, *PD* Progressive Disease, *DCR* Disease Control Rate.

PD-1 monoclonal antibody is consistently administered following traditional chemotherapy as first-line treatment in the clinical setting. However, in second-line treatment or beyond, the therapeutic response to PD-1 monotherapy has been explored in 11 studies, with only one examining the clinical efficacy of PD-1 monoclonal antibody plus gemcitabine. This Phase II study of nibuzumab plus gemcitabine was conducted across seven centers of the Korean Clinical Research Group^20^. All participating patients with R/M-NPC received nibulizumab and gemcitabine intravenously every two weeks, with cycles repeated every 28 days for up to one year. Compared to previous studies on anti-PD-1/PD-L1 monotherapy, the combination of nibulizumab and gemcitabine demonstrated improved PFS and OS (Table 1). The notable clinical efficacy observed may be attributed to the synergistic interaction between nibulizumab and gemcitabine. It is suggested that gemcitabine-induced apoptosis of tumor cells could enhance DCs-mediated presentation of tumor antigens to T cells, thereby augmenting the response and survival outcomes of nibulizumab in R/M-NPC^21^. Conversely, in the Phase I randomized Study of Spartalizumab versus Chemotherapy in patients with R/M-NPC, no improvement in median PFS was observed in the crossover group of patients who switched to Spartalizumab following treatment progression on chemotherapy^22^. In conclusion, whether the survival benefits for patients with R/M-NPC can be enhanced by a combination of chemotherapy and immunotherapy or by immunotherapy postchemotherapy progression remains uncertain and requires further investigation.

Histological subtypes, EBV status, and PD-L1 expression were not utilized for patient selection in any of the clinical trials. The efficacy of anti-PD-1 monoclonal antibody in R/M-NPC may vary depending on the PD-L1 expression status, which raises an important question for investigation as PD-L1 expression could potentially serve as a biomarker for treatment guidance. A systematic review and meta-analysis^23^ comprising 12 prospective trials (*n* = 1088) examined the significance of PD-L1 expression in predicting response to anti-PD-1/PD-L1 therapy in HNSCCs. The study concluded that using a 1% threshold, ORR was greater for PD-L1 expressers vs non-expressers (18.9% vs 8.8%), but not at 12 or 18 months. In addition, significant benefits were not observed in ORR for PD-L1 expressers defined at different thresholds. Therefore, while PD-L1 expression serves as a crucial consideration for immunosuppressive treatment approaches, its prognostic significance remains controversial for R/M-NPC. In the JUPITER-02 trial, patients with PD-L1-positive and -negative tumors exhibited similar median PFS (11.4 vs. 11.0 months) when treated with the toripalimab-GP combination. In the POLARIS-02 trial, PD-L1-positive patients (PD-L1 > 1%) showed a numerically higher ORR (27.1% vs 19.4%) compared to PD-L1-negative patients, and with even higher ORR (38.1% vs. 19.3%) observed in individuals with high PD-L1 expression (PD-L1 > 25%). Additionally, patients with high PD-L1 expression had improved median PFS (7.2 vs. 1.9 months). Current evidence suggests that anti-PD-1/PD-L1 therapy may provide modest benefits regardless of PD-L1 expression status. The prognostic value of PD-L1 expression level as a biomarker in predicting the immunotherapy response in R/M-NPC appears to be controversial, warranting further exploration.

In conclusion, the progress in PD-1 monoclonal antibody development has provided further clinical benefits for R/M-NPC. However, due to the significant heterogeneity within the TIME, PD-1 blockade therapy may only benefit a limited proportion of R/M-NPC patients, and there is a lack of effective biomarkers to screen the most suitable candidates. This highlights the need for novel immunotherapeutic strategies to improve patient survival^24^.

## Adoptive immune cell therapy for R/M-NPC

NPC cells express a limited repertoire of EBV proteins, predominantly EBV nuclear antigen 1 (EBNA1), with a subset of tumors also expressing latent membrane proteins 1 and 2 (LMP1 and LMP2)^25–27^. As EBV transitions into latency phase 2, most EBV proteins become transcriptionally silenced, an astute evasion tactic that impedes immune recognition. Consequently, these EBV proteins present as attractive targets for immunotherapy, as they can serve as specific antigens to stimulate the host immune system. Importantly, NPC cells possess the ability for immunologic processing, allowing recognition by cytotoxic T lymphocytes (CTLs)^28,29^. Taking inspiration from the successful use of adoptive EBV-targeted CTL (EBV-CTL) therapy in treating EBV-associated post-transplant lymphoproliferative disease^30^, anti-EBV immunotherapy strategies have gained attention as potential adjuvant treatments with the potential to improve R/M-NPC prognosis^25,31,32^. Table 2 presents the key characteristics and findings from 12 studies investigating the use of adoptive immune cell therapy to target systemic antitumor immunity in R/M-NPC.

**Table 2:** Characteristics of the included studies on adoptive immune cell therapy for R/M NPC

Study	Study design	Population	Sample Size	Age (range)	Male (%)	Immune preparations	CR	PR	SD	PD	ORR/DCR (%)	PFS (months)	OS (months)
Lin 2002	Phase I study	R/M NPC	16	47.6 (36–57)	13/81.2%	LMP2-DCs →CD8^+^CTLs	NS	NS	NS	NS	12.5%	NS	NS
Comoli 2005	Phase I-II study	R/M NPC	10	41.6	10/100%	EBV-CTL		2	4	4	20%	6 (4-15)	NS
Louis 2010	Phase I/II clinical trial	R/M NPC	23	29.2 (11–63)	17/73.9%	EBV-CTL	5	2	3	5	30.4%	35.3 1-year PSF rate, 65% 2-year PFS rate, 52%	1-year OS rate, 87% 2-year OS rate, 70%
Chia 2011	Prospective, open-labeled single-center phase II trial	M-NPC	16	49.7 (36–58)	13/81%	Ad-ΔLMP1-LMP2 DC vaccine	NS	1	2	13	18.8% (DCR)	1.92 (95%CI:1.6-2.1)	6.0 (95%CI:2.73-11.7)
Chia 2013	Phase 2 clinical trial	R/M NPC	35	57 (27–77)	26/73.7%	GP-CTL	2	13	7	0	42.9%	7.6 (95% CI:7.4-8.4)	1-year OS rate, 77.1% 2-year OS rate, 62.9% 3-year OS rate, 37.1%
Li 2012	Clinical trial	M-NPC	GP + CIK arm, 30 GP arm, 30	48.6 (29–58) 45.6 (33–62)	26/86.7% 24/80%	CIK + GP GP	3/0	18/14	2/3	2/13	70% (21/30) 46.7% (14/21)	26.00 ± 2.69 19.00 ± 2.19	Undefined 23.00 ± 3.94
Lim 2012	Open-label phase I clinical trial	R/M NPC	7	48.6	6/85.7%	Cetuximab+NK cell	NS	NS	4	3	57.1% (DCR)	NS	NS
Smith 2012	Phase I clinical trial	R/M NPC	14	NS	NS	AdE1-LMPpoly CTL	NS	NS	10	4	71.4% (DCR)	4.4 (1.3-14)	17.4
Smith 2017	Prospective study	20 ARMD 9 N/MRD	29	46 (34–68) 49 (22–66)	18/90% 8/88.9%	AdE1-LMPpoly CTL	N/MRD 6	N.S	NS	N/MRD 3	ARMD 60.0% (DCR)	5.5 (95% CI:2.1-9.0)	38.1 (95%CI:17.2-NR)
Li 2015	Retrospect study	M-NPC	GP + CIK arm, 112 GP arm, 110	44.6 (32–63) 45.3 (33–62)	83/74.1% 85/77.3%	CIK + GP	NS	NS	NS	NS	NS	21 1-year, 2-year, 3-year PFS rates of 76.0%, 32.1%, and 23.8%, res[ectively 5 1-year, 2-year, 3-year PFS rates of 70.0%, 24.5%, and 17.0%, respectively	32 year, 2-year, 3-year OS rates of 90.2%, 65.2%, and 25.9%, respectively 23 1-year, 2-year, 3-year OS rates of 85.5%, 47.3%, and 19.1%, respectively
Huang 2017	Phase 1/2 trial	R/M NPC	21	48.4	18/85.7%	EBV-CTL	1	NS	NS	20	4.8%	2.2	16.7
E N 2021	Phase I trial	R/M NPC	12	58	5/41.6%	CD 137 L-DC-EBV-VAX	0	1	4	7	42.0% (DCR)	16.5 (3-136)	90.5 (10-161)

*NS* Not Stated, *R/M NPC* recurrent and metastatic nasopharyngeal carcinoma, *ARMD* active recurrent/metastatic disease, *N/MRD* no or minimal residual disease, *ORR* Overall Response Rate, *CR* Complete Response, *PR* Partial Response, *SD* Stable Disease, *PD* Progressive Disease, *DCR* Disease Control Rate.

### EBV-specific cytotoxic T lymphocyte therapy

EBV-CTL therapy has emerged as a promising avenue in the pursuit of more effective treatment for R/M-NPC. The safety and efficacy of this approach have been investigated in several clinical investigations, offering valuable insights into its potential benefits.

Smith et al.^33^ pioneered the use of AdE1-LMPpoly, a vector based on polyepitopes, in a phase I clinical trial. This adenovirus-based vector encodes several CTL epitopes derived from LMP1 and LMP2 fused to a truncated EBNA1, in the absence of an intrinsic glycine-alanine repeat sequence^34,35^. Expanded EBV-specific T lymphocytes were observed in 16 (72.7%) of the 24 NPC patients. Grade I and/or II toxicities were the only adverse effects identified with the administration of AdE1-LMPpoly-expanded T cells by infusion, suggesting its safety. Among the 14 patients who received T-cell treatment, 10 maintained stable disease and showed an extended PFS (median, 66.5 days). Further analysis of a larger NPC patient cohort^36^, including both pre-emptive and therapeutic treatments, emphasized that the stabilization of the disease in patients with active recurrent/metastatic disease was significantly associated with the functional and phenotypic composition of T cell immunotherapy expanded in vitro.

Huang et al.^37^ conducted a phase 1/2 trial involving the administration of EBV-CTLs to 21 patients with R/M-NPC. Only one patient with metastatic disease achieved a complete response, resulting in an ORR of only 4.8%. A low incidence of severe adverse events was observed. However, two patients showed renewed responses to gemcitabine following EBV-CTL immunotherapy. These findings, together with previous study results, suggest that EBV-CTL immunotherapy could potentially serve as a primer for or be combined with chemotherapy^34,38^. To enhance the efficacy of EBV-specific T cells, future efforts can involve exploring combination treatments with other immune modulators, such as checkpoint inhibitors or drugs that target regulatory T cells^37^. Furthermore, upcoming studies can investigate the possibility of renewed responses to chemotherapy in patients who have previously showed no response to either chemotherapy or immunotherapy.

### Combination of chemotherapy with adoptive cell therapy

As previously noted, the clinical efficacy of EBV-CTL-based adoptive immunotherapy varies. However, when combined with the gemcitabine, carboplatin, and paclitaxel chemotherapy regimen, its therapeutic response and OS rates rank among the highest for palliative regimens^33,38^. Chia et al. conducted a phase 2 trial that paved the way for the investigation of its potential use in combination with chemotherapy as a first-line treatment^39^. In this study, 35 patients received CTLs following chemotherapy. The findings were encouraging, with two patients (5.7%) achieving complete response, 13 (31.7%) experiencing partial response, and seven (20%) maintaining stable disease as the best response to CTL therapy. This combined approach resulted in an impressive clinical benefit rate of 62.9% and a response rate of 42.9%. Notably, among these patients receiving both chemotherapy and CTLs (GP-CTL), the median PFS and OS were 7.6 months (95% CI: 7.4–8.4) and 29.9 months (95% CI: 20.8–39.3), respectively, surpassing the median PFS observed during the CTL immunotherapy phase alone (3.7 months; 95% CI: 2.4–4.0).

Transfusing autologous cytokine-induced killer cells (CIKs) represents another form of adaptive cell transfer. CIKs are co-induced with various cytokines, including anti-CD3 monoclonal antibody, IL-2, and IFN-γ. These cells exhibit robust antitumor effects because they co-express the markers for T cells (CD3) and natural killer cells (CD56)^40,41^. Both in vitro and in vivo studies have shown that their tumor-killing activity, which is restricted by non-major histocompatibility complex (MHC), to be effective against a wide range of tumor types. Li et al.^42^ utilized a retrospective cohort of 222 patients to evaluate the efficacy of GP followed by CIK. Their analysis of long-term efficacy revealed significantly higher rates of PFS and OS in the GP + CIK group. However, Fumet et al.^43^ showed that chemotherapy drugs have a dual effect, that is, they directly eliminate tumor cells and enhance their susceptibility to immune responses. While chemotherapy offers numerous benefits, it can also impair functional immune cells, thereby compromising immunity. Conversely, CIK immunotherapy, involving the artificial transfusion of functional immune cells, can achieve a combined effect that maximizes the benefits of both chemotherapy and immunotherapy. These findings bolster the concept that CIK and GP synergize to enhance antitumor efficacy.

### Dendritic cells-targeting vaccine

DCs play a pivotal role in activating anti-tumor T cells and other functions that bridge innate and adaptive immunity^44–48^. Their primary function involves capturing, processing, and presenting exogenous antigens to T lymphocytes, characterized by the constitutive expression of MHC-I and costimulatory molecules. One of the most important steps in developing CD8 T cell immunity against tumors is cross-priming, in which DCs activate CD8 T cells by cross-presenting exogenous antigens^7^. Moreover, unlike other antigen-presenting cells, DCs may deliver tumor antigens to draining lymph nodes, where they can trigger the activation of T cells^44–48^. Tumor-resident DCs are emerging as key regulators of the T-cell response within tumors during therapy^44,49–51^, establishing DCs as the hub of the anti-tumor T-cell response and prompting the development of DC-based vaccines.

Currently, only a limited number of DC-based immunotherapies have been developed to target EBV antigens associated with NPCs. Given the scarce treatment options available for R/M-NPC, investigating DC vaccines to harness the antitumor immune capabilities of DCs holds promise in both immunological and clinical efficacy. Various approaches have been investigated in the development of DC vaccines. The most common method involves the ex vivo expansion of DCs derived from monocytes (Fig. 1). Initially, peripheral blood monocytes are isolated from the apheresis product. These cells are then cultured with IL-4 and granulocyte-macrophage colony-stimulating factor to induce their differentiation into DCs. Following this, a maturation cocktail is applied to stimulate the DCs, which are subsequently pulsed with relevant tumor-associated antigens or tumor lysates, enabling them to elicit tumor-specific immune responses^52^.

In 2021, Nickles et al.^53^ conducted a groundbreaking clinical trial utilizing EBV antigens pulsed into CD137 ligand (CD137L)-DC for R/M-NPC. Antigen-presenting cells express CD137L, which is crucial for costimulating CD137-expressing T cells. This innovative approach involves reverse CD137L signaling, inducing the differentiation of monocytes into CD137L-DC, a novel subtype of dendritic cells derived from monocytes. Notably, CD137L-DCs exhibit an increased capacity for T-cell stimulation. Approximately 33.3% (4/12) of the patients reported adverse effects of grade 1, indicating that the intervention was well-tolerated. Encouragingly, clinical benefit was observed in 42% of cases, including one patient achieving partial remission and four patients experiencing stable disease for 2 to 3 years. The median PFS was 16.5 weeks (ranging from 3 to 136 weeks). These study findings are particularly promising as they demonstrate a correlation between clinical benefit and T cell responses, particularly memory and effector T cells.

## Discussion

R/M-NPC is a highly heterogeneous spectrum of disorders with various subtypes, such as de novo metastasis, locoregional recurrence, and locoregional recurrence with distant metastasis^54^. In recent years, immunotherapy has demonstrated manageable safety profiles and substantial benefits for different types of NPC.

Studies have shown that additional locoregional radiation therapy for patients with de novo metastatic NPC can significantly increase OS from 13.0-24.5 to 21.0-60.0 months^55–60^. Locoregionally recurrent NPC presents a different challenge, whereby patients have the option to undergo re-radiotherapy or salvage surgical intervention. Salvage intensity-modulated radiotherapy is the most commonly preferred treatment modality for this group. Some studies have demonstrated that combining radiotherapy with PD-1 monoclonal antibodies can achieve higher efficacy for R/M-NPC, suggesting that radiotherapy may augment the response rates to ICIs by creating a more favorable TIME^61,62^.

For R/M-NPC, palliative systemic chemotherapy remains the mainstream treatment option, typically involving platinum-containing regimens. Notably, results from the pivotal CAPTAIN-1st trial have garnered the approval of the National Medical Products Administration for the employment of the camrelizumab plus GP combination as a first-line therapeutic option for patients with R/M-NPC^63^. Afterward, following the results of the POLARIS-02 trial, toripalimab was granted approval for the management of patients with R/M-NPC who exhibited inadequate response to second-line or subsequent-line systemic therapy^63^. This implies that the addition of anti-PD1 antibodies into the standard palliative treatment regimen for R/M-NPC patients could potentially improve their OS.

Adoptive immune cell therapy, mainly targeting DCs, is well-tolerated in patients with NPC and has shown promising immune responses and clinical benefits. As previously mentioned, the traditional method for preparing DC vaccines involves culturing peripheral blood mononuclear cells with IL-4 and granulocyte-macrophage colony-stimulating factor. This process induces their differentiation into DCs, followed by stimulation of their maturation and pulsing with relevant tumor-associated antigens or tumor lysates. The administration route for DC vaccines varies; however, intranodal injection of DCs has demonstrated the ability to induce a more potent T cell anti-tumor immunity compared to intravenous or subcutaneous injection^64^. However, there are some uncertainties in the detection of vaccine-induced anti-tumor responses^65^. First, it has been noted that the amplification of adoptive CTL in peripheral blood is not as significant as observed in vitro. Second, no significant correlation has been established between CTL amplification and clinical efficacy.

Recently, Wen et al.^66^ introduced an innovative approach to tumor treatment whereby they used a nanoVaccine to induce the formation of intratumor tertiary lymphoid structures. This nanoVaccine contains the antigen EBNA1Δ93‑236, cytosine-phosphate-guanine, and Mn2+ formulated through physical interaction. T cells, DCs, and B cells are activated via the TLR-9 and STING signaling pathways by Mn2+ and cytosine-phosphate-guanine in a synergistic manner. In comparison to conventional NPC vaccinations, which primarily target T cell activation, this innovative nanoVaccine stimulates the development of tertiary lymphoid structures. NanoVaccine administered via subcutaneous injection can target and deliver antigens and adjuvants to the lymph nodes, activating robust innate and adaptive immunity. More importantly, this approach facilitates the infiltration of anti-tumor immunocytes into tumor sites, creating a favorable environment for interaction with the tumor microenvironment. By using this approach, it is possible to overcome the constraints associated with current therapeutic vaccinations and increase the population that may benefit from immunotherapy.

## Challenges and prospects

According to our pooled analysis in supplementary materials, the ORR for anti-PD1 inhibitor monotherapy was 23% when it was used as a second-line and beyond treatment. The clinical benefits obtained from the application of immune checkpoint blockades are not as ideal compared to other solid tumors. Numerous theories have been advanced about the mechanisms that underlie ICI resistance. One theory suggests that inadequate anti-tumor responses, resulting from immune evasion, may hinder the effectiveness of immunotherapies. Resistance to ICIs could be attributed to insufficient neoantigens and evasion of specific tumor molecules targeted by ICIs^67^. Additionally, reduced expression of MHC-I/MHC-II molecules may impede antigen presentation and contribute to ICIs resistance^68^. Another theory posits that immunotherapies can be rendered unsuccessful due to inadequate antitumor effector T cell activity. Impairment of the therapeutic response could result from the suppression of critical immunological signaling pathways due to the absence of downstream immune signals, including IFN-γ and IL-12^50^. However, IL-10 released by macrophages or other immunosuppressive cells, as well as tumor-derived factors like Vascular endothelial growth factor, can inhibit the maturation of conventional dendritic cells and directly inhibit their production of IL-12^8^.

Similarly, the limited efficacy of DC-targeting vaccines may be attributed to systemic immunosuppression factors, such as the increased frequency of inhibitory regulatory T cells and the presence of immunosuppressive cytokines like IL-10 or transforming Vascular endothelial growth factor-β in the peripheral blood. Additionally, the local TIME can induce immunosuppression, posing a significant challenge to cancer immunotherapy^69–71^. Indeed, the TIME-mediated regulation and suppression of tumor-infiltrating DCs might hinder their ability to initiate potent antitumor immunity and even promote tumor progression, as suggested by accumulating data. Overcoming tumor-induced immunosuppression represents a major hurdle in cancer immunotherapy. Consequently, extensive research is underway to elucidate the mechanisms by which DCs modulate anti-tumor CD8 T cell responses.

Building upon the existing achievements and addressing the unmet needs, future works and development of immunotherapy strategies for improving anti-tumor immune activity could focus on the following directions: 1) Understanding molecular mechanisms: Investigating the molecular mechanisms underlying the interactions between various immune cells and nasopharyngeal carcinoma cells to identify more targetable EBV antigens and enhance their immunogenicity; 2) Promoting immune migration: Exploring strategies to enhance the migration of peripheral immune effector cells to lymphatic organs or tumor sites and regulating the TIME; and 3) Combination therapies: Investigating the potential benefits of combining standard-of-care therapies with novel immunotherapies, which holds promise for improving treatment efficacy.

Identifying the ideal biomarker to effectively stratify R/M-NPC patients who could benefit from immunotherapy remains a significant challenge. However, there is consensus that metrics such as the the degree of early increase in EBV DNA titer and baseline plasma EBV DNA levels hold promise in forecasting long-term outcomes for R/M-NPC patients undergoing immunotherapy^72^. Other commonly used predictive biomarkers in immunotherapy include the expression of PD-L1, tumor mutation burden, and MHC-I/MHC-II gene expression^73^. These biomarkers have also been highlighted in the prognostic assessment of adoptive immune cell therapy.

However, due to the complexity of the TIME and the immune system, it is unlikely that a single biomarker alone can reliably predict prognosis and response to immunotherapy^74^. Instead, there is growing interest in exploring artificial intelligence (AI)-based approaches to integrate multi-omics data, such as genomics, pathomics, radiomics, and TIME heterogeneity, to define novel meta-biomarkers. AI techniques have been applied to various cancers, primarily non-small cell lung cancer, to discover predictive biomarkers for the efficacy of ICIs^75^. The application of AI in NPC studies could help identify more suitable patient populations for immunotherapy.

## Conclusion

Immunotherapy, particularly ICIs, has proven its therapeutic efficacy in the treatment of R/M-NPC. Furthermore, adoptive immune cell therapy has exhibited promising therapeutic potential and merits continued investigation in clinical settings. Delving into the mechanisms governing the interaction between systemic immunity, local tumor immunity, and the TIME is anticipated to enhance anti-tumor activity and uncover additional targets for immunotherapy. Furthermore, the utilization of AI to analyze predictive biomarkers and identify appropriate patient cohorts for immunotherapy holds promise as a future advancement.

### Supplementary information

Supplementary materials

## Data Availability

As this is a review, there are no original datasets. However, all referenced data sources are cited within the manuscript.

## Code availability

As this is a review, there are no codes generated.
