# Supplementary material for: Immunotherapy for recurrent or metastatic nasopharyngeal carcinoma
Source: NPJ Precis Oncol. 2024 May 16;8:101. doi: 10.1038/s41698-024-00601-1 (PMC11099100; doi:10.1038/s41698-024-00601-1)
Supplement: Supplementary file 1 — Supplementary materials [file 41698_2024_601_MOESM1_ESM.pdf]

Supplementary material

Methods

Search strategy

A thorough search was undertaken across several databases, such as Embase, Cochrane, PubMed, and the Web of Science, to obtain pertinent research articles concerning the efficacy of immunotherapy in R/M-NPC. Our analysis incorporated cross-sectional studies, prospective and retrospective cohort studies, and randomized controlled trials (RCTs). Supplementary Material provides a comprehensive description of the search strategy. To ensure methodological rigor, we carefully removed studies with a high risk of bias. Two authors (Xin Liu and Hui Shen), each reviewed article abstracts separately to make sure these articles were eligible for inclusion in the study. In cases of discrepancies or uncertainties during the screening process, a third independent author (Bin Zhang) was consulted to reach a consensus and resolve any divergent assessments.

Eligibility criteria

The following constituted the inclusion criteria: 1) Research objects: Studies focusing on patients with R/M-NPC. 2) Clinical Studies: Studies that provided data on the ORR of immunotherapy in R/M-NPC. 3) Clinical outcomes: Studies that reported relevant clinical outcomes, including the DCR, OS, and PFS, as well as the rate of adverse events graded according to the severity associated with the use of immunotherapy for R/M-NPC. 4) Full-text studies: Only full-text research articles were considered for inclusion. 5). Peer-reviewed journals: We exclusively included studies published in peer-reviewed journals. 6). Study design: Both single-arm and controlled studies were eligible for inclusion.

To ensure the accuracy of our analysis, we implemented the following exclusion criteria: 1) Animal Studies: Studies conducted on animals were excluded from our consideration. 2) Case Reports: Individual case reports were not taken into account. 3) Reviews: Comprehensive reviews were not included in our analysis. 4) Letters: Correspondence in the form of letters was not incorporated. 5) Conference Proceedings: Studies presented as conference proceedings were excluded. 6) Abstracts: Abstracts were not incorporated into our analysis.

Data extraction

The relevant data from the included publications were meticulously extracted into a standardized proforma by two impartial authors (Xin Liu and Hui Shen). The data that were retrieved included a wide range of patient and study details, such as study location, design, sample size, demographic information (age and sex), treatment arms, dosage regimen, and previous treatment records. ORR was the primary outcome. Secondary outcomes consisted of DCR, OS, PFS, and the rate of adverse events graded by severity.

Evaluation of publication bias and risk of bias

During the meta-analysis phase, two independent reviewers (Xin Liu and Hui Shen) conducted a blinded assessment of the quality of both non-randomized studies and RCTs utilizing the Risk of Bias in Non-Randomized Studies of Interventions (ROBINS-I) tool and the Cochrane Risk-of-Bias Tool for Randomized Trials (RoB 2) tool, respectively. Each study was rigorously evaluated based on seven key aspects: selection of reported results, outcome measurement, participant selection, missing data, intervention classification, deviations from intended interventions, and confounding. Based on these evaluations, each study was categorized into one of four categories according to its level of bias: low, moderate, serious, or critical, providing an overall assessment of the study's bias risk. Visual inspection of funnel plot asymmetry and execution of Egger's test were carried out to evaluate publication bias.

statistical analysis

The "meta" package in R Studio (Version 4.0.3) was utilized for all analyses of statistical data. For the meta-analysis, ORR, DCR, and adverse events were pooled as proportions using a random-effects model. $P<0.05$ was set as the significance criterion. We used the I2 statistic and Cochran's Q test values to assess statistical heterogeneity among the studies included. According to conventional definitions, low, moderate, and high degrees of heterogeneity were denoted by I2 values of 25%, 50%, and 75%, respectively. Additionally, significant heterogeneity was indicated by a Cochran's Q test result with a $P\leq 0.10$.

Identified studies

After removing duplicates, a total of 848 articles were extracted in the initial search. Among them, the inclusion criteria for the meta-analysis were satisfied by 12 studies comprising 740 patients **(Supplementary Figure 1)**. summary of the essential characteristics of the included articles is provided in **Table 1** and **Table 2**, respectively. The study designs varied, with four adopting a retrospective approach and eight employing a prospective perspective. Based on the evaluation conducted using the ROBINS-I tool, three of the eleven non-randomized included studies were found to exhibit a low risk of bias, whereas the other eight were classified as having a moderate risk of bias **(Supplementary Table 2)**. The risk of bias in the included RCTs was determined to be moderate using the RoB 2 tool **(Supplementary Table 3)**.

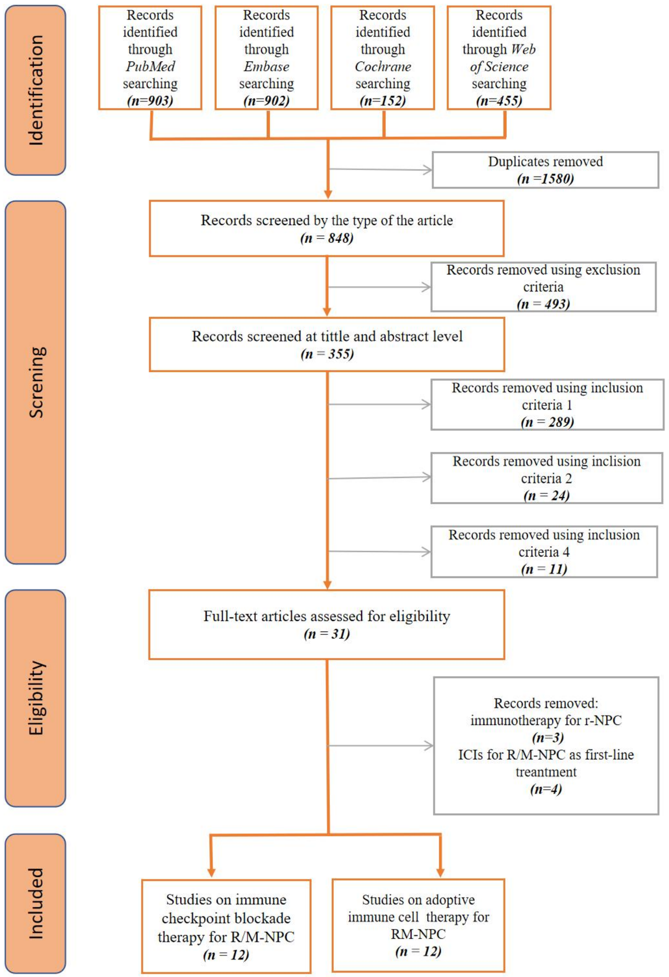

Supplementary Figure 1. PRISMA flow diagram of the study selection process. Abbreviation: r-NPC: recurrent nasopharyngeal carcinoma.

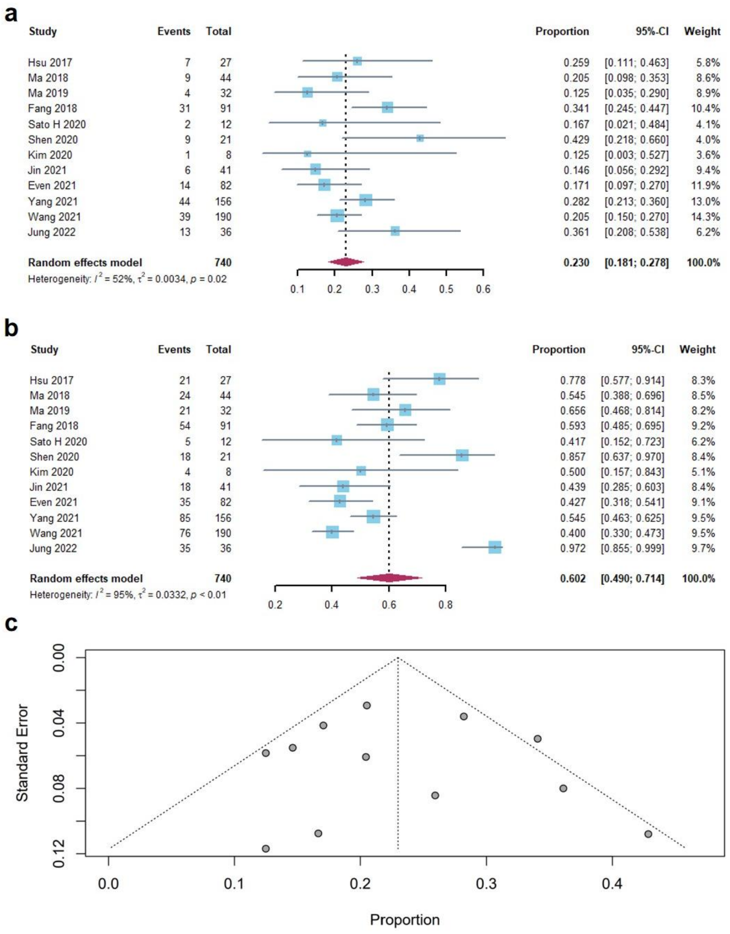

Supplementary Figure 2. Forest plots and funnel plot. (a) Forest plot illustrates the pooled overall response rates of anti-PD1 inhibitors in the second-line and beyond treatment of R/M-NPC; (b) Forest plot shows the pooled disease control rates of anti-PD1 inhibitors in the second-line and beyond treatment of R/M-NPC; (c) Funnel plot indicates the overall response rates of anti-PD1 inhibitors in the second-line and beyond treatment of R/M-NPC.

Supplementary Table 1. Search strategy

**PubMed**	
**#1**	("Nasopharyngeal Neoplasms"[Mesh] OR NPC[tw]) OR (("Nasopharyngeal Diseases"[MeSH] OR "Nasopharynx"[Mesh] OR nasophar* OR rhinophar*[tw] OR naso-phar*[tw] OR chonae[tw]) AND ("Neoplasms"[Mesh] OR carcinom*[tw] OR cancer*[tw] OR precancer*[tw] OR pre-cancer*[tw] OR neoplas*[tw] OR tumor*[tw] OR tumour*[tw] OR malignan*[tw] OR premalignan*[tw] OR pre-malignan*[tw]))
**#2**	(immunotherapy[MeSH Terms]) OR (immunother*[Title/Abstract]) OR (Cancer vaccines[MeSH Terms]) OR (vaccin*[Title/Abstract]) OR (immunisation[Title/Abstract]) OR (immunization[Title/Abstract])
**#3**	#1 AND #2
**EMBASE**	
**#1**	'nasopharyngeal carcinoma':ab,ti OR 'nasopharyngeal cancer':ab,ti OR npc:ab,ti
**#2**	('immunotherapy':ab,ti OR 'immunother*':ab,ti OR 'cancer vaccine':ab,ti OR 'vaccin*':ab,ti OR 'immunisation':ab,ti OR 'immunization':ab,ti)
**#3**	#1 AND #2
**Web of Science**	
**#1**	TS =('nasopharyngeal cancer' OR 'nasopharyngeal carcinoma' OR NPC)
**#2**	TS = (immunotherapy OR immunother* OR cancer vaccine OR vaccin* OR immunisation OR immunization)
**#3**	#1 AND #2
**Cochrane Library**	
**#1**	('nasopharyngeal cancer' OR 'nasopharyngeal carcinoma' OR NPC)
**#2**	(immunotherapy OR immunother* OR cancer vaccine OR vaccin* OR immunisation OR immunization)
**#3**	#1 AND #2

Supplementary Table 2. Risk of Bias Assessment Using the Risk Of Bias In Non-Randomised Studies of Interventions (ROBINS-I) Tool

Study	Bias due to Confounding	Bias of Selection of Participants	Bias in Classification of Interventions	Bias due to Deviations from Intended Interventions	Bias due to Missing Data	Bias in Measurement of Outcomes	Bias in Selection of The Reported Result	Overall Bias
Hsu, 2017	Low	Low	Low	Low	Low	Low	Low	Low
Fang, 2018	Low	Low	Low	Low	Low	Moderate*	Low	Moderate
Ma, 2018	Low	Low	Low	Low	Low	Moderate*	Low	Moderate
Ma, 2019	Low	Low	Low	Low	Low	Moderate*	Low	Moderate
Kim, 2020	Low	Low	Low	Low	Low	Moderate*	Low	Moderate
Sato H, 2020	Low	Low	Low	Low	Low	Moderate*	Low	Moderate
Shen, 2020	Low	Low	Low	Low	Low	Moderate*	Low	Moderate
Jin, 2021	Low	Low	Low	Low	Low	Moderate*	Low	Moderate
Wang, 2021	Low	Low	Low	Low	Low	Moderate*	Low	Moderate
Yang, 2021	Low	Low	Low	Low	Low	Low	Low	Low
Jin,2022	Low	Low	Low	Low	Low	Low	Low	Low

*Assessors were not blinded

Supplementary Table 3. Risk of Bias Assessment Using Cochrane Risk-of-Bias Tool for Randomized Trials (RoB 2)

**Study**	**Bias Arising from the Randomization Process**	**Bias due to Deviations from Intended Interventions**	**Bias due to Missing Outcome Data**	**Bias in Measurement of Outcomes**	**Bias in Selection of Reported Results**	**Overall Bias**
Even, 2021	Moderate	Moderate	Low	Low	Low	Moderate
